# Identification of circRNA Expression Profiles in BMSCs from Glucocorticoid-Induced Osteoporosis Model

**DOI:** 10.1155/2022/3249737

**Published:** 2022-02-04

**Authors:** Zhipeng Chen, Wei Lin, Shengli Zhao, Xiaoyi Mo, Zhenxing Wen, Wing Hoi Cheung, Dan Fu, Bailing Chen

**Affiliations:** ^1^Department of Spine Surgery, The First Affiliated Hospital of Sun Yat-sen University, Guangzhou, China; ^2^Department of Orthopedics, Sun Yat-sen Memorial Hospital, Sun Yat-sen University, Guangzhou, China; ^3^Guangdong Provincial Key Laboratory of Orthopedics and Traumatology, The First Affiliated Hospital of Sun Yat-sen University, Guangzhou, China; ^4^Department of Orthopaedics and Traumatology, Prince of Wales Hospital, The Chinese University of Hong Kong, Hong Kong SAR, China; ^5^Department of Orthopedics, Kiang Wu Hospital, Macau, China

## Abstract

**Background:**

Circular RNAs (circRNAs) contribute to the regulation of many diseases. However, little is known about the role of circRNAs in the development of glucocorticoid-induced osteoporosis (GIOP). The present study is aimed at systematically characterizing the circRNA expression profiles in GIOP and predict the potential functions of the associated regulatory networks.

**Methods:**

A small animal GIOP model was developed in Sprague-Dawley rats given daily intraperitoneal doses of the synthetic glucocorticoid dexamethasone. Micro-CT and bone histomorphometry were performed to characterize the bone loss. Alizarin red S (ARS) staining and alkaline phosphatase (ALP) activity were assessed to determine the osteogenic differentiation potential of BMSCs. RNA sequencing was performed to identify differentially expressed circRNAs in BMSCs between the GIOP and normal groups, which were validated by qRT-PCR. siRNA interference experiments were used to demonstrate their function. Gene Ontology (GO) and Kyoto Encyclopedia of Genes and Genomes (KEGG) analyses were performed to predict the functions of differentially expressed circRNAs. The microRNA (miRNA) targets of the circRNAs and circRNA-miRNA interactions were predicted.

**Results:**

Micro-CT and bone histomorphometry confirmed the rat GIOP model. Both ARS intensity and ALP activity were decreased in GIOP BMSCs. Seventeen circRNAs were identified by fold change = 2.0, *p* < 0.05, and false discovery rate < 0.05, of which 7 were upregulated and 10 were downregulated. The qRT-PCR results of the selected circRNAs were consistent with the RNA-seq results and showed that circARSB and circAKT3 were significantly upregulated, while circPTEN and circTRPM7 were downregulated in the GIOP group. Further functional experiments found that downregulation of circARSB and circPTEN expression resulted in a corresponding change in osteogenic differentiation, suggesting that circARSB negatively, while circPTEN positively, regulates BMSC osteogenic differentiation. Analysis of circRNA-targeted miRNAs predicted that miR-135a-5p was associated with circARSB and circAKT3, and miR-881-3p was associated with circPTEN and circTRPM7. Furthermore, the signalling pathways associated with these differentially expressed circRNAs were predicted.

**Conclusions:**

The present study identified circARSB, circAKT3, circPTEN, and circTRPM7 as being associated with osteogenic differentiation during GIOP through a circRNA-targeted miRNA-mRNA axis, which might provide insight into the pathophysiological mechanism of GIOP.

## 1. Introduction

Glucocorticoid-induced osteoporosis (GIOP), the most common type of secondary osteoporosis, is one of the most common complications after long-term administration of glucocorticoids (GCs). It occurs in 30% patients with long-term (over 6 months) use of GC [1]. It has been reported that low bone mineral density (BMD) and subsequent fragility fractures are associated with the development of GIOP [2–4], seriously affecting the quality of life of patients and increasing the economic burden.

Mesenchymal stromal cells (MSCs) are well known for their self-renewal ability to differentiate into multiple cell types in vitro and in vivo including osteogenic, chondrogenic, adipogenic, and myogenic lineages [5–7]. The most common source of MSCs is bone marrow MSCs (BMSCs), which are commonly used in skeletal tissue engineering *in vitro* and *in vivo* [8]. BMSCs have previously been demonstrated to play a role in disease progression and treatment responses and have been used for bone regeneration [9]. Previous studies reported that long-term use of external GCs was associated with dysfunction of BMSCs and therefore resulted in impaired osteogenesis and increased osteoblast apoptosis, all of which led to a reduction in bone formation and a decrease in bone mass [10, 11]. Although the understanding of pathogenesis of GIOP has made great progress, and the terminal differentiation of BMSCs are known to be tightly modulated by diverse transcription factors and various signalling pathways [12, 13], the specific mechanisms have not yet been fully elucidated, and further researches are needed [14]. Therefore, there is an urgent need for a better understanding of the pathogenesis of GIOP to identify novel biomarkers and develop new strategies for the prevention and treatment of GIOP.

Circular RNAs (circRNAs), a new class of noncoding RNAs, form covalently closed loops and represent a more stable and widespread class of RNA molecules than linear RNAs [15]. circRNAs play an important role in biological processes and serve as diagnostic and prognostic biomarkers of neural development [16], cancers [17], and cardiovascular diseases [18], among others. Besides, it has been reported that circRNAs also exert their functions in orthopaedic diseases, including osteosarcoma [19], osteoarthritis [20], and disc degeneration [21]. However, the circRNA profile and their role in bone regeneration of GIOP remain unknown.

In the present study, we aimed to identify differentially expressed circRNAs in BMSCs between the GIOP group and the normal group using RNA sequencing (RNA-seq) and to study their regulatory interaction networks and underlying mechanism based on bioinformatics analysis. These findings may help elucidate the specified mechanism of GIOP development and reveal novel diagnostic biomarkers for GIOP.

## 2. Materials and Methods

### 2.1. GIOP Animal Model Preparation

A total of ten Sprague-Dawley healthy female rats aged 4 months with an average weight of 269.2 ± 2.434 g were obtained from the Guangdong Experimental Animal Centre. The rats were randomly assigned equally into two groups, and the grouping and dosing were as follows: normal group, 5 ml/kg distilled water; GIOP group, 5 mg/kg dexamethasone. Both treatments were administered by intraperitoneal injection twice a week for 12 consecutive weeks. After that, the rats were sacrificed by cervical dislocation. The femurs were removed and placed in aseptic PBS for further extraction of BMSCs. The left and right tibias were removed by dissection and placed in 4% paraformaldehyde prior to assessment of bone morphometry. Lumbar vertebra-4 was dissected and placed in 4% paraformaldehyde for further microcomputed tomography (micro-CT) analysis. All animal treatment methods were performed in accordance with the relevant standards of the Ethics Committee of the First Affiliated Hospital of Sun Yat-sen University (2019-196).

### 2.2. Micro-CT

Lumbar vertebra-4 from the normal and GIOP groups was analysed by a high-resolution micro-CT scanner (Hitachi-Aloka, Japan). The scanner was set at a voltage of 55 kV, a current of 145 mA, and a resolution of 10 *μ*m per pixel. Cross-sectional images of the lumbar vertebra-4 were used to perform three-dimensional histomorphometric analysis by imaging reconstruction software (VGStudio MAX V2.2). The analysed three-dimensional structural parameters included BMD, number of trabecular bone (Tb. N), trabecular thickness (Tb. Th), bone volume/total volume (BV/TV), and trabecular separation (Tb. Sp).

### 2.3. Bone Histomorphometry

The bone tissues were fixed in 4% paraformaldehyde, decalcified by ethylenediamine tetraacetic acid solution (EDTA) with 10% formalin for 4 weeks, and then, embedded in paraffin. The paraffin-embedded tibia tissues were sectioned into 4 *μ*m-thick specimens for staining with haematoxylin-eosin. The stained tibia was observed and imaged with a light microscope (Olympus BX43, Tokyo, Japan). All measurements were performed with an image analysis system (Olympus cellSens Dimension, Tokyo, Japan). All parameters were measured according to the guidelines set by the American Society of Bone Mineral Research Histomorphometry Nomenclature Committee (1987) [22].

### 2.4. Extraction of BMSCs and Osteogenic Differentiation Cultures

BMSCs were isolated and cultured following the whole bone marrow adherence method. The femurs were removed and placed in a small aseptic beaker and then moved to a super clean bench. After washing with PBS, the bilateral mummification ends in the femur were resected. A syringe was used to aspirate the cell culture fluid to flush the bone marrow cavity, which was repeated 3-5 times. The cells were resuspended in Dulbecco's modified Eagle's medium: nutrient mixture F-12 (DMEM/F12; Gibco) supplemented with 10% foetal bovine serum (FBS; Gibco), 100 IU/ml penicillin, and 100 IU/ml streptomycin, after which the cells were seeded into flasks and cultured in an incubator at 37°C, 5% CO_2_, and 100% relative humidity. After 48 hours, the fluid was changed to remove the suspended cells. Thereafter, the medium was changed every 3 days. When BMSCs reached 80-90% confluence, 0.25% trypsin containing 0.53 mM EDTA was used to digest the cells. The BMSCs were then expanded and used for *in vitro* and *in vivo* experiments at passages 3-5.

BMSCs were seeded in 12-well plates at a density of 1.5∗10^4^cells/cm^2^ in growth medium (GM) consisting of DMEM/F12 and 10% FBS. When the culture reached 80% confluence, the medium was changed to osteogenic differentiation medium (OM, Cyagen Biosciences), which was replaced every 3 days. Alkaline phosphatase (ALP) activity and alizarin red S (ARS) staining were used to determine the osteogenic differentiation potential after induction on days 7 and 14, respectively. For ARS quantification, 10% cetylpyridinium chloride monohydrate (CPC, Sigma-Aldrich) was used to destain the cells for 1 hour at room temperature. Then, 200 *μ*l of liquid was transferred to a 96-well plate, and spectrophotometric absorbance was measured at 562 nm.

### 2.5. RNA Extraction and Sequencing

BMSCs from the two groups were harvested after 5 days of osteogenic induction. Total RNA was isolated with TRIzol reagent (Invitrogen). rRNAs were removed using Ribo-Zero rRNA Removal Kits (Illumina). The RNA purity and concentration of the samples were determined by a NanoDrop ND-1000 (NanoDrop, Thermo). The RNA integrity of the samples was determined by denaturing agarose gel electrophoresis.

RNA libraries were constructed by rRNA-depleted RNAs with the TruSeq Stranded Total RNA Library Prep Kit (Illumina). The libraries were quality controlled and quantified with the BioAnalyzer 2100 system (Agilent Technologies). Next, 10-pM libraries were denatured as single-stranded DNA molecules, captured on Illumina flow cells, amplified in situ as clusters, and finally sequenced on an Illumina NovaSeq 6000 Sequencer with 150 bp paired end reads.

### 2.6. Quantitative Real-Time Polymerase Chain Reaction (qRT-PCR) Analysis

To quantify the expression of the selected circRNAs, total RNA (2 *μ*g) was subjected to first-strand cDNA synthesis with dNTP Mix (HyTest Ltd.), RNase inhibitor (Enzymatic), and SuperScript III Reverse Transcriptase (Thermo Fisher Scientific). qRT-PCR was performed on a QuantStudio 5 Real-Time PCR System (Thermo Fisher) using SYBR Green master mix (Cloudseq). ACTB was used for normalization. The primer sequences were as follows: circARSB, forward: 5′-CTCTGGAACAACACGGTCCT-3′, reverse: 5′-TACATTCCCAGGTGCCATTT-3′; circAKT3, forward: 5′-TGGTTCGAGAGAAGGCAAGT-3′, reverse: 5′-TTGGCTTTGGTCGTTCTGTT-3′; circTRPM7, forward: 5′-GCACAGAAGCTCACATTTGC-3′, reverse: 5′-TGGGAGAACTCTCCTCCAGA-3′; circPTEN, forward: 5′-GAGGCCCTGGATTTTTATGG-3′, reverse: 5′-GCAGTTAAATTTGGCGGTGT-3′; ACTB, forward: 5′-AAGTCCCTCACCCTCCCAAAAG-3′, reverse: 5′-AAGCAATGCTGTCACCTTCCC-3′. The relative expression was calculated by the formula 2^−ΔΔCt^. Each sample was tested in triplicate in three independent experiments.

### 2.7. siRNA Transfection Assay

Transfection was conducted when cells reached 70–80% confluence. The si-circARSB, si-circPTEN (Supplementary 2), and corresponding negative controls were transfected separately using Lipofectamine 3000 (Thermo Fisher, USA) according to the manufacturer's protocol. The cells were collected 48 h after transfection for mRNA analysis by qRT-PCR.

### 2.8. Statistical Analysis

Quantitative data are expressed as the means ± standard deviation (SD), and all experiments were performed at least three times. The statistical analysis was performed with SPSS 20.0 software. The differences between two groups were analysed by unpaired *t*-test, while one-way analysis of variance (ANOVA) was utilized to identify the differences between more than two groups. A *p* < 0.05 was considered statistically significant.

## 3. Results

### 3.1. Growth Status of Rats

Before treatment, rats in two groups exhibit increased no significant differences in overall body weight (269.7 ± 8.0 and 268.6 ± 9.6 g in GIOP group and normal group, *p* = 0.83). After 12 weeks of intervention, the body weight of rats in the GIOP group was significantly lower than that in the normal group (352.5 ± 19.4 vs. 549.5 ± 35.9, *p* < 0.0001).

### 3.2. Verification of GIOP Rat Model

**Figure 1:**
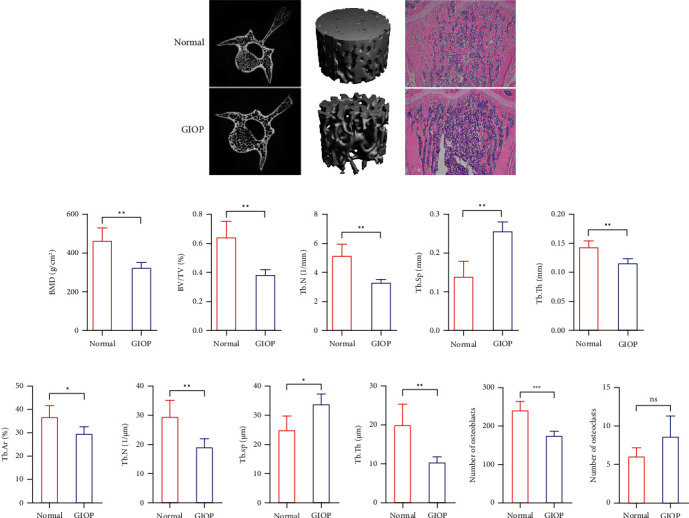
Micro-CT analysis of the lumbar vertebra and HE staining of tibial sections of saline-treated and dexamethasone-treated rats (a). Low BMD, increased separation, and loss of trabeculae were observed after dexamethasone treatment compared with the saline control through micro-CT (b) and HE analysis (c). The number of osteoblasts decreased significantly in the GIOP group (d).

A three-dimensional reconstruction of the region of interest (ROI), 2D sectional images of the lumbar vertebrae, and HE staining of tibial sections in the normal and GIOP groups were shown in Figure 1(a). Micro-CT analyses were used to evaluate the alteration of the trabecular bone microarchitecture of lumbar vertebrae in rats, and BMD, BV/TV, Tb. N, Tb. Th, and Tb. Sp of trabecular bone in lumbar vertebrae decreased significantly in the GIOP group (*p* < 0.05) (Figure 1(b)). Histomorphometric analyses were initially performed using bones from the tibia. The histomorphometric data of the tibia were provided in Figure 1(c). Compared with those in the normal group, the Tb. Ar%, Tb. N, Tb. Th, and Tb. Sp decreased significantly in the GIOP group (*p* < 0.05). What is more, we found that compared with that in the normal group, the number of osteoblasts decreased significantly in the GIOP group (*p* < 0.001), while there were no significant differences in the number of osteoclasts between groups (Figure 1(d)).

### 3.3. The Osteogenic Effects of GC in BMSCs

**Figure 2:**
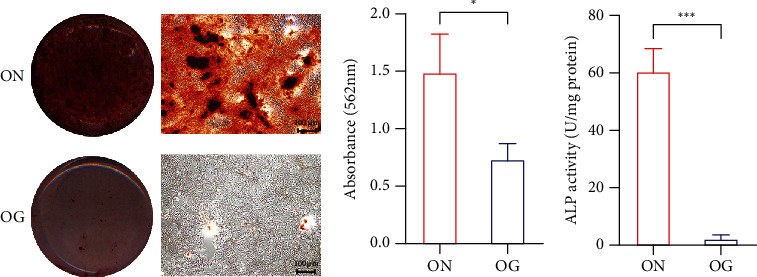
Representative images (a) and the quantification (b) of ARS staining of BMSCs isolated from normal and GIOP rats and cultured in differentiation medium for 14 days. The quantification of ALP activity showing the difference of BMSCs differentiated for 7 days (c).

After 7 days and 14 days of osteogenic induction, the ARS staining results indicated that mineralized nodules were increased in the osteogenic induction of normal BMSCs group (ON) than in the osteogenic induction of GIOP BMSCs group (OG) (Figure 2(a)). And the result of quantification of ARS was consistent with the result of ARS staining (Figure 2(b)). In addition, the results of ALP activity confirmed reduced ALP activity of GIOP BMSCs during osteogenic differentiation (Figure 2(c)).

### 3.4. Expression Patterns of circRNAs

**Figure 3:**
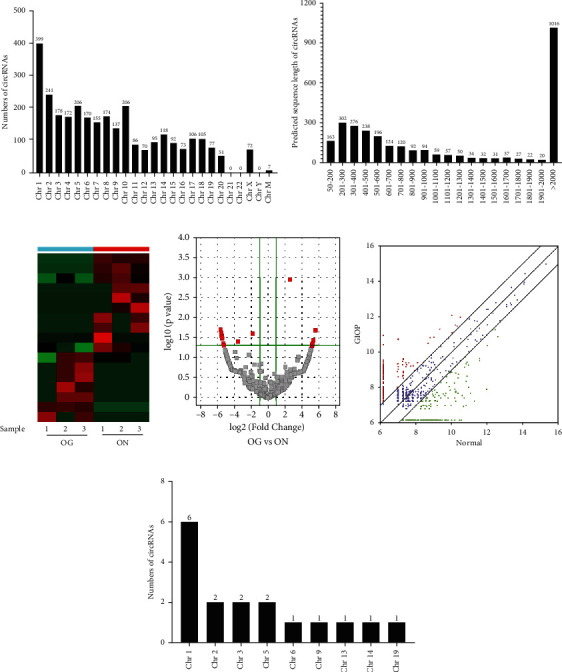
The profile of circRNAs in the OG and ON groups. Distribution of circRNAs in chromosomes (a); “ChrM” represents the mitochondrial genome. The length distribution of 2990 circRNAs (b); the majority (33.63%) were 201-500 nt in size. A heat map was generated to assess the differentially expressed circRNAs between OG and ON (c). Volcano plot and scatter plot showing that 10 circRNAs were downregulated and 7 circRNAs were upregulated during osteogenesis (d, e). The distribution of differentially expressed circRNAs on chromosomes (f).

In total, 2990 circRNAs were identified in BMSCs after GC treatment. Among these, 1081 circRNAs have been recorded in circBase and/or reported by other studies, while the remaining 1909 circRNAs were novel circRNAs in this study. The 2990 circRNAs were distributed across almost all chromosomes. Chromosomes 1-20 comprised 2911 circRNAs, X contained 72 circRNAs, and 7 circRNAs were found in mitochondrial chromosomes (Figure 3(a)). The size of 1695 exonic circRNAs ranged from 62 nt to 81015 nt, and the majority of them (33.63%) were 201-500 nt long, with an average length of 2480.41 nt (Figure 3(b)). An overview of differentially expressed circRNAs between the GIOP group and the normal group is displayed by a heat map after fold change filtering (Figure 3(c)). In total, volcano plot and scatter plot for circRNAs showed that 17 circRNAs were identified to be differentially expressed, among which 7 circRNAs were upregulated and 10 circRNAs were downregulated significantly (fold change ≥ 2.0, *p* < 0 .05) (Figures 3(d) and 3(e)). And the specific information of those differentially expressed circRNAs was shown in Table 1. Overall, 11, 4, and 2 of them belonged to the exon type, sense overlapping type, and intergenic region type, respectively. And the distribution of differentially expressed circRNAs on chromosomes was shown in Figure 3(f).

**Figure 4:**
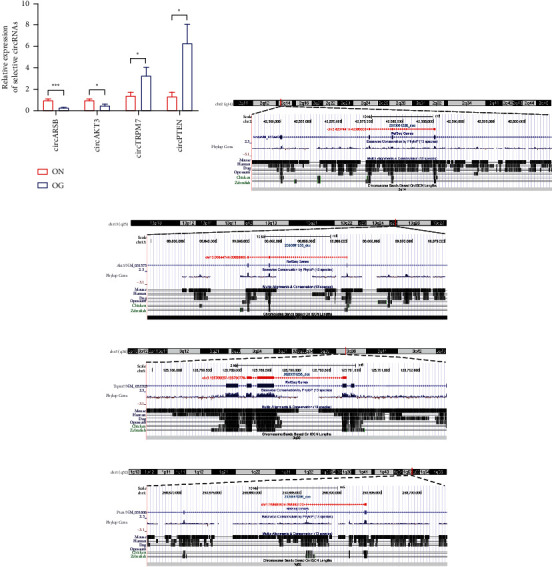
qRT-PCR was used to examine the expression of circARSB, circAKT3, circTRPM7, and circPTEN during the osteogenic induction of normal BMSCs and GIOP BMSCs (a). The schematic diagram showed the genomic locus of circARSB (b), circAKT3 (c), circTRPM7 (d), and circPTEN (e) in ARSB, AKT3, TRPM7, and PTEN gene.

Among them, four circRNAs, namely, circARSB (chr2:42574415-42585308+), circAKT3 (chr13:99644745-99660065-), circTRPM7 (chr3:125789087-125790770-), and circPTEN (chr1:258685624-258693123+), were selected based on their raw intensities, fold changes, and *p* values. circARSB and circAKT3 were upregulated, while circTRPM7 and circPTEN were downregulated in the GIOP group by qRT-PCR. The validation results suggested that the expression levels of these four selected circRNAs were consistent with the RNA-seq results (Figure 4(a)). According to the UCSC Genome Browser, all of these circRNAs are exonic type. To be specific, circARSB is formed by the circularization of exon 2, exon 3, and exon 4 of the Arsb gene on chromosome 2q14 and ultimately formed the mature sequence with a length of 586 nt (Figure 4(b)); circAKT3 is formed by the circularization of exon 3, exon 4, and exon 5 of the Akt3 gene on chromosome 13q25 and ultimately formed the mature sequence with a length of 389 nt (Figure 4(c)); circTRPM7 is formed by the circularization of exon 33, exon 34, and exon 35 of the Trpm7 gene on chromosome 3q36 and ultimately formed the mature sequence with a length of 433 nt (Figure 4(d)); circPTEN is formed by the circularization of exon 3, exon 4, and exon 5 of the Pten gene on chromosome 1q52 and ultimately formed the mature sequence with a length of 328 nt (Figure 4(e)).

### 3.5. Knockdown of circARSB and circPTEN Regulates Osteogenic Differentiation of BMSCs

Transfection was conducted to knock down the expression of circARSB and circPTEN. qRT-PCR analysis confirmed that the expression of circARSB and circPTEN was decreased in the knockdown groups (Figure S1(a)). The activity of ALP was decreased in the circARSB knockdown groups while increased in the circPTEN knockdown groups after induction for 7 days (Figure S1(b)). Following osteogenic induction for 14 days, the intensity of ARS staining was significantly decreased in the circARSB knockdown groups while increased in the circPTEN knockdown groups, indicating that matrix mineralization was decreased (Figure S1(c)).

### 3.6. GO and KEGG Pathway Analyses of the Host Genes of circRNAs

**Figure 5:**
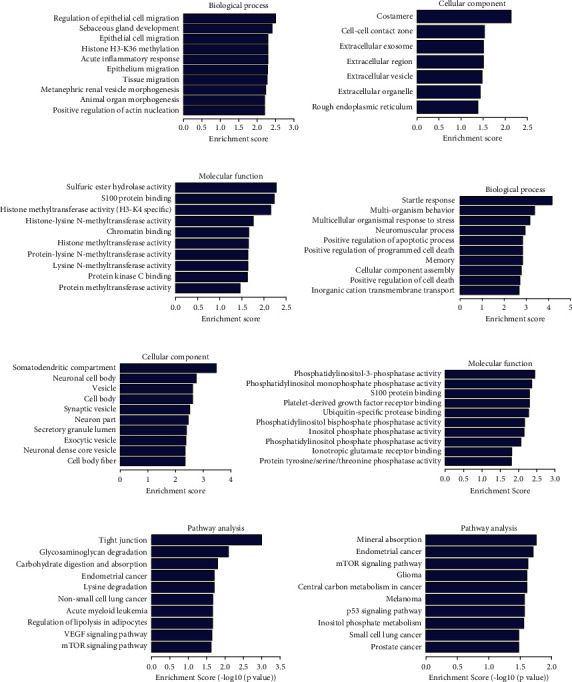
GO analyses and KEGG pathway analysis: enrichment map of GO analyses of upregulated circRNAs: biological process, cellular component, and molecular function (a, b, c); enrichment map of GO analyses of downregulated circRNAs: biological process, cellular component, and molecular function (d, e, f); enrichment map of KEGG pathway analysis of upregulated circRNAs (g); enrichment map of KEGG pathway analysis of downregulated circRNAs (h).

circRNAs can affect the osteogenic differentiation process of GIOP BMSCs by regulating corresponding signaling pathways, which may involve in development of GIOP. Therefore, we performed GO enrichment analyses to explore the potential function of differentially expressed circRNAs. Gene Ontology (GO) analysis consists of three aspects: biological processes, cellular components, and molecular functions. Analysis of the top 128 enriched GO terms associated with upregulated circRNAs indicated that the most enriched biological process terms (top) were associated with sebaceous gland development (GO:0048733), negative regulation of acute inflammatory response (GO:0002674), and histone H3-K36 methylation (GO:0010452). The most enriched cellular component terms were costamere (GO:0043034), cell-cell contact zone (GO:0044291), and extracellular exosome (GO:0070062). The most enriched molecular function terms were related to sulfuric ester hydrolase activity (GO:0008484), S100 protein binding (GO:0044548), and histone methyltransferase activity (H3-K4 specific) (GO:0042800) (Figures 5(a)–5(c)).

According to analysis of the top 340 enriched GO terms associated with downregulated circRNAs, the most enriched biological process terms were associated with positive regulation of apoptotic process (GO:0043065), positive regulation of programmed cell death (GO:0043068), and positive regulation of cell death (GO:0010942). The most enriched cellular component terms were somatodendritic compartment (GO:0036477), neuronal cell body (GO:0043025), and vesicle (GO:0031982). The most enriched molecular function terms were related to phosphatidylinositol-3-phosphatase activity (GO:0004438), phosphatidylinositol monophosphate phosphatase activity (GO:0052744), and platelet-derived growth factor receptor binding (GO:0005161) (Figures 5(d)–5(f)).

For the Kyoto Encyclopedia of Genes and Genomes (KEGG) pathway analysis of upregulated circRNAs, the top 73 pathways are listed according to the enrichment scores, and specific pathways related to osteogenic differentiation and antiadipogenic differentiation were enriched, including regulation of lipolysis in adipocytes, VEGF signalling pathway, apoptosis, and mTOR signalling pathway. For the downregulated circRNAs, the top 21 pathways are listed according to the enrichment scores, and specific pathways related to osteogenic differentiation and antiadipogenic differentiation were enriched, including mineral absorption, mTOR signalling pathway, FoxO signalling pathway, and PI3K-Akt signalling pathway (Figures 5(g) and 5(h)).

### 3.7. Prediction and Annotation of miRNA Targets of circRNAs

**Figure 6:**
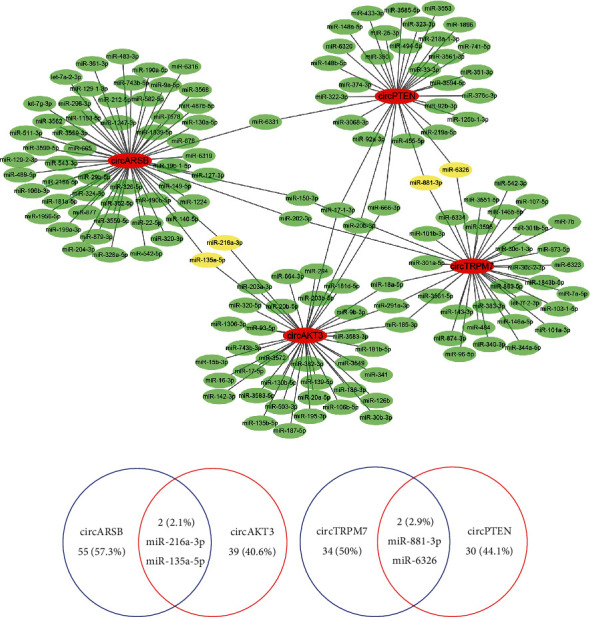
The relationship between differentially expressed circRNAs and their target miRNAs (a). The cotargeted miRNAs of circARSB and circAKT3 (b), circTRPM7, and circPTEN (c).

The circRNA-miRNA networks of the selected circRNAs (Table 2) were predicted by miRanda and mapped by Cytoscape (Figure 6(a)). Next, we analysed the target miRNAs of these four circRNAs and found that miR-216a-3p and miR-135a-5p could be targeted by both circARSB and circAKT3 in the network (Figure 6(b)). For circPTEN and circTRPM7, miR-881-3p and miR-6326 could be targeted by both circRNAs in the circRNA-miRNA network (Figure 6(c)). We hypothesized that these four circRNAs act as miRNA sponges to regulate the circRNA-miRNA network, and their predicted interactions suggested that they might play a crucial role in the process of GIOP development.

## 4. Discussion

To characterize GC-induced bone loss in a small animal model, we investigated the effects of the synthetic GC dexamethasone in rats, which has been developed as the most commonly used model for osteoporosis, based on micro-CT, histomorphometry, and imaging methodologies [23, 24]. In this study, we showed that the most significant change detected in the bones of rats treated with dexamethasone is a decrease in bone formation, as measured in trabecular bone. In other words, changes in several parameters of trabecular bone, such as BMD, Tb. Ar%, BV/TV, BS/BV, Tb. N, Tb. Th, and Tb. Sp, were detected by micro-CT and histomorphometric analysis. In addition, it has been reported that GC exerts a negative influence on osteoblast differentiation and proliferation, but significantly increased osteoblastic apoptosis [25]. Since new bone formation is mainly dependent on the differentiation of BMSCs into osteoblast lineage, the negative effect of high glucocorticoid on BMSCs will undoubtedly result in bone loss in GIOP. In this study, we found that the number of osteoblasts was reduced in GIOP rats, while the number of osteoclasts between GIOP rats and normal rats was not statistically different, which indicated that GIOP-related pathologies in the GIOP rats could be a consequence of induced osteoblasts.

Noncoding RNAs, such as miRNAs, circRNAs, and lncRNAs, regulate various processes at the RNA level and are involved in numerous diseases, including GIOP. In fact, there is only a few literatures studied the relationship between noncoding RNA and GIOP, most of which focus on the relationship between miRNAs and the occurrence and development of GIOP. For example, the study of Ren et al. [26] analyzed the miRNA expression in human with GIOP through high-throughput sequencing and found six significantly upregulated miRNAs and three significantly downregulated miRNAs, which provide novel insight into the mechanism of GIOP and lay a good foundation for the prevention and treatment of GIOP. Moreover, there are a few additional researches associated with specific molecular. Shen et al. [27] found that let-7f-5p promoted Dex-inhibited osteoblast differentiation and prevented Dex-induced bone loss in vitro and in vivo by targeting TGFBR1, which subsequently mediates the downstream osteogenic transcription as a potential therapeutic target for GIOP. A study by Ma et al. [28] found that miR-186 can be used as a therapeutic approach in the management of glucocorticoid-induced bone loss by mediating the suppression of cathepsin K. A study of Larson and Satterthwaite [29] demonstrated that miR-365 could inhibit DXM-induced osteoclastogenesis through the suppression of RANKL expression in osteoblasts and directly regulate the bone resorption of osteoclasts. A study from Liu et al. [30] demonstrated that miR-106b expression increased in C57BL/6 mice with GIOP. Besides, miR-106b negatively regulated osteogenic differentiation of mesenchymal stem cells in vitro partly through directly targeting BMP2. In conclusion, the association between miRNAs and GIOP has attracted attention; however, investigations focusing on the association between alterations of circRNAs expression in GIOP are limited.

It has been reported that circRNAs can act as miRNA sponges and play an essential regulatory role through interactions with disease-related miRNAs [31] in several orthopaedic diseases, including ankylosing spondylitis [32] and osteoporosis [33]. For example, in patients with osteoporosis, Zhao et al. [34] identified circ_0001275 as a potential diagnostic biomarker in postmenopausal osteoporosis; Huang et al. [35] performed microarray analysis and PCR validation, which showed that circ_0006873 and circ_0002060 were associated with a low BMD state. In patients with osteosarcoma, circTada2a was reported to promote the development of osteosarcoma and increase tumor malignant behavior through miR-203a-3p/CREB3 axis, which was expected to be a new therapeutic target for osteosarcoma [19]. In osteoarthritis, circserPine2 may provide a potentially effective treatment strategy for osteoarthritis by reducing chondrocyte apoptosis and promoting extracellular matrix anabolism through the miR-1271/ERG pathway [20]. In intervertebral disc degeneration, circRNA-CIDN was revealed to promote the homeostatic of extracellular matrix through miRNA-34a-5p/SIRT1 axis and inhibit apoptosis, providing a new perspective for the study of the pathogenesis of compression intervertebral disc degeneration [21]. However, the transcriptome expression profiling of GIOP, especially the specific functions of circRNAs during the osteogenic differentiation of BMSCs in GIOP, is rarely reported [36, 37]. In our study, we sequenced all circRNAs obtained from BMSC samples from GIOP rats and normal controls and identified 2990 circRNAs, including 17 circRNAs that were differentially expressed by a *p* value < 0.05 and fold change > 2.0. Among them, 7 circRNAs were upregulated, while the other 10 were downregulated in the process of osteogenesis in GIOP BMSCs. Four circRNAs, including circARSB, circAKT3, circTRPM7, and circPTEN, were selected based on their raw intensities, fold changes, and *p* values and were validated by qRT-PCR. circARSB and circAKT3 were confirmed to be significantly upregulated, while circTRPM7 and circPTEN were downregulated in the GIOP BMSC samples. According to the bioinformatics analysis, the host genes of circARSB, circAKT3, circTRPM7, and circPTEN, namely, Arsb [38, 39], Akt3 [40, 41], Trpm7 [42, 43], and Pten [44, 45], respectively, have been implicated as important regulators of bone metabolism. In our in-depth research, the downregulation of circARSB and circPTEN expression resulted in a corresponding change in osteogenic differentiation, suggesting that circARSB negatively, while circPTEN positively, regulates BMSC osteogenic differentiation.

The roles of circRNAs in osteogenesis may be related to miRNA-mediated effects. miRNAs, a class of small noncoding RNA sequences, are essential for the posttranscriptional regulation of gene expression [46]. According to previous studies, miRNAs play crucial roles in regulating osteogenic differentiation. Chen et al. [47] confirmed that miR-19a-3p promotes the osteogenic differentiation of hMSCs by inhibiting HDAC4 expression, thus alleviating the progression of osteoporosis. Cheng et al. [48] revealed that miR-365a-3p negatively regulates the osteogenic differentiation of hBMSCs by targeting RUNX2, thus promoting the progression of osteoporosis. Based on our hypothesis that circARSB, circAKT3, circTRPM7, and circPTEN may function as potent miRNA sponges for their predicted miRNA binding sites, we predicted the functions of differentially expressed circRNAs and the potential related mechanism. miR-216a-3p and miR-135a-5p could be targeted by both circARSB and circAKT3 in the circRNA-miRNA network. For circTRPM7 and circPTEN, miR-881-3p and miR-6326 could be targeted by both circRNAs in the circRNA-miRNA network. In previous studies, miR-135a-5p was reported to play critical roles in the development of osteoporosis. For example, Chen et al. [49] demonstrated that miR-135a-5p suppressed 3T3-L1 preadipocyte differentiation and adipogenesis through the activation of canonical Wnt/*β*-catenin signalling by directly targeting Apc. Gao et al. [50] confirmed that miR-135a-5p played an active role in adipogenesis by targeting LATS1 and MOB1B expression, thereby promoting the HIPPO signalling pathway. Shi and Zhang [51] found that miR-135a-5p may play a role in osteoporosis progression by regulating osteogenic differentiation via RUNX2. For miR-216a-3p, however, there have been no studies about its role in bone metabolism. Here, miR-135a-5p was predicted to be the target of circARSB and circAKT3 in the circRNA-miRNA network during the process of osteogenic induction. miR-881-3p was identified in certain insulin secretion and age-related functional pathways in aged nonalcoholic fatty liver disease rats [52] and was found to be differentially expressed in the hippocampi in response to chronic high-dose alcohol administration in rats [53]. However, there is no previous study about the role of miR-6326 in bone metabolism. We hypothesized that miR-881-3p, together with circTRPM7 and circPTEN, might play an important role in the circRNA-miRNA network in promoting the osteogenic differentiation of BMSCs in GIOP.

Although we identified the differentially expressed circRNAs in rats with GIOP, the underlying mechanism is still poorly understood. We hypothesized that miR-135a-5p could be targeted by both circARSB and circAKT3 in the circRNA-miRNA network and that miR-881-3p could be targeted by circTRPM7 and circPTEN in the circRNA-miRNA network. Therefore, we performed GO and KEGG pathway analyses to predict the functions of differentially expressed circRNAs and potentially related mechanisms. In the present study, we found that several pathways of bone metabolism in the enrichment analysis were associated with circRNAs that were upregulated in GIOP. These pathways include regulation of lipolysis in adipocytes, VEGF signalling pathway, and mTOR signalling pathway, which are considered to play important roles in bone metabolism [54–58]. Similarly, pathways of bone metabolism associated with circRNAs downregulated in GIOP were found, including mineral absorption, mTOR signalling pathway, FoxO signalling pathway, and PI3K-Akt signalling pathway. The results suggested that these circRNAs might play important roles in regulating the physiological processes of GIOP. Therefore, we propose that the circRNA-miRNA-mRNA axis may be involved in the mechanism of promoting osteoblast differentiation. However, further research is needed to validate this mechanism.

In summary, we reported the circRNA expression profile in GIOP rats by RNA-seq analyses. Related regulatory interaction networks were constructed based on the sequencing data. These original discoveries could provide some clues for the biological functions of circRNAs in the pathophysiological mechanism of GIOP. Furthermore, the mechanism and function of the differentially expressed circRNAs should be further verified by strict molecular biological experimental research and should be studied deeply in future work.

## 5. Conclusions

Our research describes the MSC circRNA expression profiles and functional networks involved in the regulation of GIOP. Our results provide possible molecular mechanisms for the development of GIOP. We hypothesized that circARSB/circAKT3-miR-135a-5p and circPTEN/circTRPM7-miR-881-3p could be the possible candidate mechanisms. These proposed mechanisms may help to elucidate possible molecular therapeutic targets for GIOP.

## Figures and Tables

**Table 1:** The differentially expressed circRNAs ranked by their RNA-seq results.

circRNA ID	circRNA type	Locus	*p* value	Log_2_ FC
*Upregulated circRNAs*				
chr1:232138805-232148068+	Sense overlapping	chr1	1.372∗10^−7^	7.5470
chr5:79444533-79448528-	Exonic	chr5	0.0206	5.6229
circARSB	Exonic	chr2	0.0366	5.3489
circAKT3	Exonic	chr13	0.0437	5.2632
chr3:111563809-111567731+	Exonic	chr3	0.0470	5.2259
chr2:207700606-207729912+	Exonic	chr2	0.0497	5.1965
chr6:137274621-137292892+	Sense overlapping	chr6	0.0011	2.6235
*Downregulated circRNAs*				
chr5:21834978-21835133-	Sense overlapping	chr5	0.0196	-5.5534
chr19:50369748-50371831+	Intergenic	chr19	0.023867	-5.46765
chr9:88377072-88400815+	Exonic	chr9	0.0246	-5.4527
chr1:228117553-228117771-	Intergenic	chr1	0.0281	-5.3943
circTRPM7	Exonic	chr3	0.0303	-5.3608
chr14:43212130-43221970+	Exonic	chr14	0.0328	-5.3251
circPTEN	Exonic	chr1	0.0436	-5.1899
chr1:181594948-181599334-	Exonic	chr1	0.0491	-5.1317
chr1:255194723-255196140-	Exonic	chr1	0.0392	-3.4921
chr1:232142345-232145992+	Sense overlapping	chr1	0.0247	-1.7518

Note: circRNA ID: the circRNA ID found in circBase (http://circbase.mdc-berlin.de). circRNA type: circRNAs were classified into five types: “exonic,” “intronic,” “antisense,” “intragenic,” and “intergenic.” *p* value: *p* value was calculated from paired *t*-test. FDR: FDR was calculated from Benjamini-Hochberg FDR. Fold change: the absolute ratio (no log scale) of normalized intensities between the two conditions.

**Table 2:** Target miRNAs of the selected circRNAs.

circRNAs	Targeted miRNAs				
circARSB	miR-6331	miR-216a-3p	miR-320-3p	miR-665	miR-326-5p
circAKT3	miR-666-3p	miR-185-3p	miR-664-3p	miR-135a-5p	miR-16-3p
circPTEN	miR-741-5p	miR-3068-3p	miR-666-3p	miR-3553	miR-92b-3p
circTRPM7	miR-30c-2-3p	miR-301b-5p	miR-383-3p	miR-881-3p	miR-146b-5p

## Data Availability

The RNA-seq data used to support the findings of this study are included within the article and are available from the corresponding author upon request.

## Supplementary Materials

Supplementary Materials Supplementary 1. Figure S1: circARSB knockdown inhibited osteogenic differentiation, while circPTEN knockdown promoted osteogenic differentiation. Knockdown efficiency of circARSB and circPTEN was evaluated by qRT-PCR assay (a). Knockdown of circARSB decreased the osteogenic differentiation capability of BMSCs, while knockdown of circPTEN increased the osteogenic differentiation capability of BMSCs, which was assessed by ALP activity analysis and ARS staining (b, c). Supplementary 2: RNA sequences of circARSB and circPTEN small interfering RNA (siRNA) oligonucleotides utilized in this study were shown as below.
