# Supplementary material for: Identification of circRNA Expression Profiles in BMSCs from Glucocorticoid-Induced Osteoporosis Model
Source: Stem Cells Int. 2022 Feb 4;2022:3249737. doi: 10.1155/2022/3249737 (PMC8837445; doi:10.1155/2022/3249737)
Supplement: Supplementary Materials — Supplementary 1. Figure S1: circARSB knockdown inhibited osteogenic differentiation, while circPTEN knockdown promoted osteogenic differentiation. Knockdown efficiency of circARSB and circPTEN was evaluated by qRT-PCR assay (a). Knockdown of circARSB decreased the osteogenic differentiation capability of BMSCs, while knockdown of circPTEN increased the osteogenic differentiation capability of BMSCs, which was assessed by ALP activity analysis and ARS staining (b, c). Supplementary 2: RNA sequences of circARSB and circPTEN small interfering RNA (siRNA) oligonucleotides utilized in this study were shown as below. [file 3249737.f1.docx]

Supplementary 1:


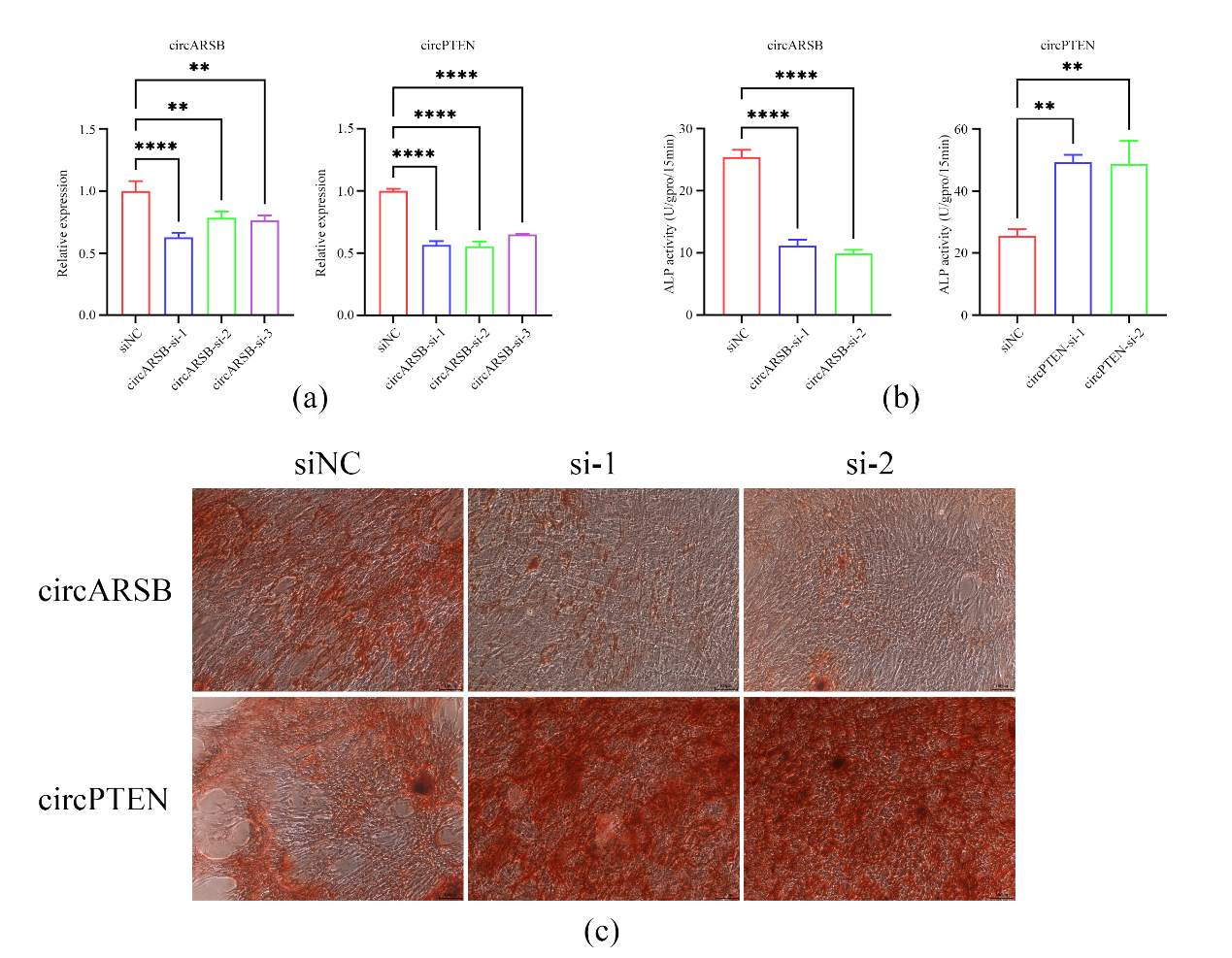


Figure S1: circARSB knockdown inhibited osteogenic differentiation, while circPTEN knockdown promoted osteogenic differentiation. Knockdown efficiency of circARSB and circPTEN was evaluated by qRT-PCR assay (a). Knockdown of circARSB decreased the osteogenic differentiation capability of BMSCs, while knockdown of circPTEN iecreased the osteogenic differentiation capability of BMSCs, which was assessed by ALP activity analysis and ARS staining (b, c).

Supplementary 2:

RNA sequences of circARSB and circPTEN small interfering RNA (siRNA) oligonucleotides utilized in this study were shown as below.

circARSB-si-1 sense（5'-3'） GAGUGGAGCUGCUACUUAAdTdT

antisense（5'-3'） UUAAGUAGCAGCUCCACUCdTdT

circARSB-si-2 sense（5'-3'） GCGUGGUCCUGGACAACUAdTdT

antisense（5'-3'） UAGUUGUCCAGGACCACGCdTdT

circARSB-si-3 sense（5'-3'） GGCAGUGAAGACUACUACAdTdT

antisense（5'-3'） UGUAGUAGUCUUCACUGCCdTdT

circPTEN-si-1 sense（5'-3'） CCUGGAGGAUUAUCUGAUAdTdT

antisense（5'-3'） UAUCAGAUAAUCCUCCAGGdTdT

circPTEN-si-2 sense（5'-3'） GCCCAGAUGUCGUCUCAAAdTdT

antisense（5'-3'） UUUGAGACGACAUCUGGGCdTdT

circPTEN-si-3 sense（5'-3'） GGCAGGAUCCUCCAGCGAAdTdT

antisense（5'-3'） UUCGCUGGAGGAUCCUGCCdTdT

siNC sense（5'-3'） UUCUCCGAACGUGUCACGUdTdT

antisense（5'-3'） ACGUGACACGUUCGGAGAAdTdT
